# Posterior cruciate ligament research output in asian countries from 2009 - 2019: A systematic review

**DOI:** 10.1016/j.amsu.2020.09.006

**Published:** 2020-09-12

**Authors:** Sholahuddin Rhatomy, Dwikora Novembri Utomo, Heri Suroto, Ferdiansyah Mahyudin

**Affiliations:** a Doctoral Program,Faculty of Medicine, Universitas Airlangga, Indonesia; b Department of Orthopaedic and Traumatology, Faculty of Medicine, Universitas Airlangga, Indonesia

**Keywords:** Posterior cruciate ligament, PCL, Research output, Asian countries

## Abstract

**Purpose:**

This study aimed to determine the number of posterior cruciate ligament (PCL) publications performed in Asian countries and to identify factors associated with research output in this region.

**Materials and methods:**

Searches of existing academic journal articles were performed using PubMed, Google Scholar, and the Cochrane Library from January 1, 2009 until December 31, 2019.

**Results:**

A total of 265 articles were published in the last 10 years in Asian countries, with an increase in publications after 2010 and an average of 26 articles every year. More than half (70%) of the articles were published in journals with an impact factor (IF) ≥1. The majority of the publications were cohort studies (27%), followed by case reports (16%), systematic reviews/meta-analyses (2.6%), laboratory studies (1.8%), and case-control studies (1.5%). South Korea and China had the most PCL publications, and most authors were from South Korea.

**Conclusion:**

The PCL research output in Asia is low in quantity but high in quality publications, and the majority of publications come from South Korea, China and Japan, with most being cohort studies and case reports.

## Highlights

• Publication Trends.
• Posterior Cruciate Ligament.
• Over the Past 10 Years.
• PubMed.
• Asian Countries.

## 1 Introduction

The posterior cruciate ligament (PCL), which consists of the anterolateral (AL) and posteromedial (PM) bundles, is the strongest ligament in the knee joint. Some initial reports have shown good functional results with nonoperative treatment for PCL injuries. Biomechanical and clinical studies suggest a less benign natural history of PCL deficiency that results in persistent symptoms and premature osteoarthritis. Longer-term follow-up studies have also described an increasing incidence of arthritis, declining knee function, and making PCL reconstruction more widely accepted, especially with the improvement of operative techniques. Although operative indications for these injuries remain controversial, there has remained a strong interest in the literature regarding the methods of reconstruction [1].

Some controversy still remains regarding the epidemiology, diagnosis, treatment, rehabilitation programs and outcomes [1,2]. An early and accurate diagnosis of PCL rupture should be based on the history of illness, a vascular examination to exclude vascular damage, a physical examination for the assessment of PCL and collateral ligament injuries, and a radiographic examination. Once the diagnosis is established, an appropriate treatment plan should be determined according to the time of injury, rupture pattern, presence of remnants, combined collateral ligament injuries, alignment, and tibial slope [1,2].

The number of publications regarding this ligament is increasing every year worldwide. There are no data that provide information on all PCL publications, especially in Asian countries. This study aims to consolidate the information on PCL research publications in Asian countries.

## 2 Methods

### 2.1 Search strategy

Electronic systematic searches were performed using PubMed, Google Scholar, and the Cochrane Library for existing academic journal articles and reviews between January 1, 2009 and December 31, 2019. The search terms included [posterior cruciate ligament] OR [PCL]. Author affiliations were subsequently screened, and all abstracts with at least one author from any of the Asian countries, according to the PRISMA guidelines for conducting the review, were followed [3]. We have registered our study in research registry with unique identifying number review registry 975.

### 2.2 Inclusion and exclusion criteria

An article was included if the study was performed in an Asian country and if the research topic was posterior cruciate ligament, PCL or PCL reconstruction. All original studies, including laboratory studies, animal studies, cadaveric studies, observational studies, interventional studies and qualitative studies, were included and published between January 1, 2009 and December 31, 2019. We also included papers in other languages when an English abstract was available. We excluded book sections, conference presentations, abstracts, guidelines, commentaries, personal views, and studies.

### 2.3 Data collection

All abstracts were screened independently by two reviewers to determine whether they met the eligibility criteria. The full texts of eligible studies were then reviewed for eligibility again, followed by data extraction and analysis.

### 2.4 Data extraction and analysis

The data of the eligible articles were abstracted into variables such as country, year of publication, journal, study design and topic. The study design was classified based on the level of evidence and included systematic reviews and meta-analyses, randomized controlled trials (RCTs), nonrandomized controlled trial (NRCTs), cohort studies, case-control studies, cross-sectional studies, case reports, cadaveric studies, animal studies, and laboratory studies.

The published journals were divided by impact factor (IF) into low- (IF below 1) and high- (IF of 1 or above) impact journals. Information regarding the country was collected, which included the number of PCL publications.

## 3 Results

### 3.1 Study selection

**Fig. 1:**
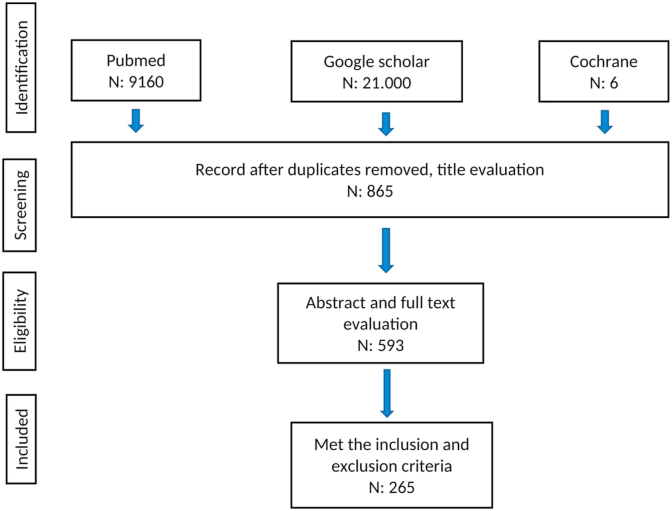
Flow diagram of the article selection process.

**The search strategy** included articles between January 1, 2009 and December 31, 2019. The search results identified 865 records. After removing duplicates and after title selection, 593 articles were selected for full-text assessment. Three hundred and twenty-eight papers were excluded for the following reasons: (1) the research topic was not posterior cruciate ligament, PCL or PCL reconstruction, (2) the study was performed outside Asia, and (3) it was not an original study, e.g., expert opinions or article replies. A total of 265 articles met the eligibility criteria for data extraction (Fig. 1).

### 3.2 Number of publications

**Fig. 2:**
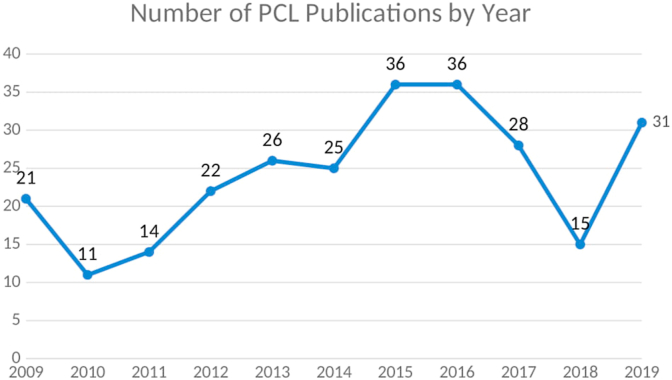
Number of PCL publication by year.

A total of 265 papers on the PCL performed in Asian countries published between 2009 and 2019 were identified (Fig. 2). The average number of published articles per year was 26. The highest numbers of publications occurred in 2015 and 2016, with 36 articles each, and the lowest number of publications occurred in 2010, with 11 articles. The total number of papers increased after 2010 and then decreased after 2016.

### 3.3 Country

Table 1 shows the top 10 countries in Asia with the most publications in the past ten years. The country with the most publications was South Korea. South Korea published 89 articles, which constituted 33% of all articles published on this topic, followed by China and Japan (30% and 11%, respectively).

**Table 1:** Top 10 country in asia with the most publications.

No	Country	Number of Publications
1	South Korea	89
2	China	81
3	Japan	29
4	India	23
5	Taiwan	12
6	Iran	9
7	Turkey	8
8	Indonesia	4
9	Malaysia	4
10	Singapore	3
11	Thailand	2
12	Saudi Arabia	1

### 3.4 Journals

Table 2 shows the top 10 journals that published articles on the PCL. The journal of *Knee Surgery, Sports Traumatology, Arthroscopy* was the leading publisher of articles on this topic, with 33 (12.4%) articles published from 2009 to 2019. The top 10 journals published 46.6% of all articles published in the past 10 years. The top 5 journals (*Knee Surgery*, *Sports Traumatology*, *Arthroscopy*; *Arthroscopy*: *The Journal of Arthroscopic & Related Surgery*; *The American Journal of Sports Medicine*; *The Journal of Knee Surgery*; *and Archives of Orthopaedic and Trauma Surgery*) contributed 35% of all articles on the PCL in the study period.

**Table 2:** Top 10 journals publishing on the PCL.

No	Journal name	Number of Publications
1	Knee surgery, sports traumatology, arthroscopy	33
2	Arthroscopy: the journal of arthroscopic & related surgery	21
3	Archives of orthopaedic and trauma surgery	18
4	Zhongguo xiu fu chong jian wai ke za zhi = Zhongguo xiufu chongjian waike zazhi = Chinese journal of reparative and reconstructive surgery	11
5	The American journal of sports medicine	10
6	Knee surgery & related research	8
7	Arthroscopy techniques	6
8	BMC musculoskeletal disorders	6
9	Zhongguo gu shang = China journal of orthopaedics and traumatology	6
10	Acta orthopaedica et traumatologica turcica	5

### 3.5 Top 10 articles with the highest number of citations

Table 3 shows the top 10 articles with the highest number of citations from 2009 to 2019. There were 9 articles reporting clinical research and 1 review article.

**Table 3:** Top 10 articles with most-number of citations.

No.	Article title	Number of citation
1	A prospective randomized study comparing arthroscopic single-bundle and double-bundle posterior cruciate ligament reconstructions preserving remnant fibers [6].	106
2	Anterolateral transtibial posterior cruciate ligament reconstruction combined with anatomical reconstruction of posterolateral corner insufficiency: comparison of single-bundle versus double-bundle posterior cruciate ligament reconstruction over a 2- to 6-year follow-up [7].	69
3	A safe postero-medial approach to posterior cruciate ligament avulsion fracture [8].	51
4	Single-tunnel suture fixation of posterior cruciate ligament avulsion fracture [9].	50
5	Arthroscopic suture fixation for avulsion fractures in the tibial attachment of the posterior cruciate ligament [10].	50
6	A comparison of arthroscopically assisted single and double bundle tibial inlay reconstruction for isolated posterior cruciate ligament injury [11].	48
7	Transtibial versus tibial inlay techniques for posterior cruciate ligament reconstruction: long-term follow-up study [12].	47
8	Rupture of posterior cruciate ligament: diagnosis and treatment principles [13].	44
9	Double-bundle posterior cruciate ligament reconstruction using a non-hardware suspension fixation technique and 8 strands of autogenous hamstring tendons [14].	43
10	Clinical comparison of conventional and remnant-preserving transtibial single-bundle posterior cruciate ligament reconstruction combined with posterolateral corner reconstruction [15].	42

### 3.6 Top 10 first authors on publications of posterior cruciate ligament research

Table 4 shows the top 10 first authors on publications of posterior cruciate ligament research. Seven were from South Korea, and 3 were from China.

**Table 4:** Top 10 first author on Posterior Cruciate Ligament Research and Publications.

No	Orthopaedic Center	Investigator	Number of Publications
1	Department of Orthopaedic Surgery, Gachon University School of Medicine, Gil Hospital, Incheon, South Korea	Lee, Yong Seuk	9
2	Department of Orthopaedic Surgery, Arthroscopy and Joint Research Institute, Yonsei University Health System, CPO Box 8044, 134 Shinchon-dong, Seodaemun-gu, Seoul 120–752, South Korea	Kim, Sung-Jae	6
3	Department of Orthopaedic Surgery, Samsung Medical Center, Sungkyunkwan University School of Medicine, Seoul, Korea	Ahn, Jin Hwan	4
4	Department of Orthopeadics, West China Hospital, Sichuan University, Chengdu 610,041, China	Chen, Gang	4
5	Department of Radiology, Kangbuk Samsung Hospital, Sungkyunkwan University School of Medicine, Seoul, South Korea	Park, Hee Jin	4
6	Center for Joint Diseases and Rheumatism, Kyung Hee University Hospital at Gangdong, Seoul, South Korea	Lee, Sang Hak	3
7	Department of Orthopaedics, Xiangya Hospital, Central South University, Changsha, Hunan Province, China	Deng, Zhenhan	3
8	Sports Medicine Service, Beijing Jishuitan Hospital, Beijing, China	Li, Yue	3
9	Department of Orthopaedic Surgery, School of Medicine, Kyung Hee University, Seoul, South Korea	Yoon, Kyoung Ho	3
10	Department of Orthopaedic Surgery, The Armed Forces Daegu Hospital, Gyeongsan, Republic of Korea	Lee, Dong-Yeong	3

### 3.7 Top 10 leading investigators on publications of posterior cruciate ligament research

Table 5 shows the top 10 investigators in all positions on publications of the PCL. Most of them were from South Korea, and 1 person was from China.

**Table 5:** Top 10 leading investigators on Posterior Cruciate Ligament Research and Publications.

No	Orthopaedic Center	Investigator	Number of Publications
1	Department of Orthopaedic Surgery, Gachon University School of Medicine, Gil Hospital, Incheon, South Korea	Lee, Yong Seuk	16
2	Department of Orthopaedic Surgery, Samsung Medical Center, Sungkyunkwan University School of Medicine, Seoul, South Korea	Ahn, Jin Hwan	10
3	Center for Joint Diseases and Rheumatism, Kyung Hee University Hospital at Gangdong, Seoul, South Korea	Lee, Sang Hak	7
4	Department of Orthopaedic Surgery, Samsung Medical Center, Sungkyunkwan University, School of Medicine,Irwon-Ro, Gangnam-gu, Seoul, South Korea	Wang, Joon Ho	7
5	Department of Orthopaedic Surgery, College of Medicine, Chung-Ang University, Seoul, South Korea	Jung, Ho-Joong	7
6	Department of Orthopaedic Surgery, Arthroscopy and Joint Research Institute, Yonsei University Health System, Shinchon-dong, Seodaemun-gu, Seoul 120–752, South Korea	Kim, Sung-Jae	6
7	Department of Orthopeadics, West China Hospital, Sichuan University, Chengdu 610,041, China	Chen, Gang	6
8	Department of Orthopaedic Surgery, Medial Center of Chung-Ang University, Knee Center, Seoul, South Korea	Jung, Young-Bok	6
9	Department of Orthopaedic Surgery, Seoul Paik Hospital, Inje University, Seoul, South Korea	Kim, Jin Goo	6
10	Department of Orthopaedic Surgery, Chung-Ang University School of Medicine, Seoul, South Korea	Lee, Han-Jun	5

Trend of publications by year and type of studies on the posterior cruciate ligament.

Table 6 shows the trend of publications by year and type of studies on the posterior cruciate ligament. The majority of the publications (27%) were cohort studies, followed by case reports (16%).

**Table 6:** Top 10 leading investigators on Posterior Cruciate Ligament Research and Publication.

Year	Molecular/Laboratory Study	Animal Study	Cadaveric Surgical Technique	Imaging Study	Surgical Technique	Case Report	Cross Sectional	Case Control	Cohort	Randomized Controlled Clinical Trial	Meta-Analysis & Systematic Review	Review Article
2009	0	0	3	1	1	4	4	0	7	0	0	1
2010	0	0	1	1	1	4	0	0	4	0	0	0
2011	1	2	2	0	0	2	1	0	3	1	0	2
2012	1	2	2	1	1	6	1	0	7	1	0	0
2013	0	0	5	0	2	3	1	2	11	0	0	2
2014	1	0	3	2	1	3	3	0	11	1	0	0
2015	1	1	5	2	3	3	9	0	7	2	1	2
2016	0	3	3	7	2	6	1	2	7	2	0	3
2017	0	0	2	3	3	6	4	0	6	0	3	1
2018	0	0	1	2	2	1	2	0	3	0	2	2
2019	1	2	1	6	2	6	1	0	8	2	1	1
Total	5	10	28	25	18	44	27	4	74	9	7	14

## 4 Discussion

The research output of PCL publications in Asian countries is low in quantity compared with that of ACL publications [4]. There have been only 265 articles in the last 10 years. This small number of publications is also suitable because the incidence of isolated posterior cruciate ligament (PCL) rupture is low among cases of knee injury [5]. South Korea and China were the countries with the most publications on the PCL (64%), followed by Japan, India and Taiwan (10%, 8% and 4%, respectively). Based on first-author and author data in all positions, South Korean authors dominated the publications on the PCL, followed by Chinese authors; additionally, Lee Yong Seuk from the Department of Orthopaedic Surgery, Gachon University School of Medicine, Gil Hospital, Incheon, South Korea is the author with the most PCL publications.

The majority of the publications (27%) were cohort studies, followed by case reports (16%), and few studies were case-control studies, laboratory studies, and systematic reviews/meta-analyses (1.5%, 1.8% and 2.6%, respectively). A subanalysis of the types of studies performed in each country showed that systematic reviews and meta-analyses were only performed in South Korea and China (1.5% and 1.1%, respectively). Laboratory studies were performed in Japan, China, and India (0.07%, 0.07%, and 0.04%, respectively). There were only a few case-control studies that were performed and published in 2 countries: Japan and China (0.07% and 0.07%, respectively).

### 4.1 Quality of the journals

The published journals were divided by impact factor (IF) into low- (IF below 1) and high- (IF of 1 or above) impact journals. A total of 186 articles published in Asian countries in the past 10 years were from high-impact journals (70%). This showed that most journals had a high quality.

One may argue that the strong motivation for publication in high-IF journals is to raise the international ranking of the universities or education centers. The other motivations are to evaluate and share new knowledge or novelty and to document the work. Twenty-four articles (9%) were conducted in Asian countries and were published in local journals in native languages, such as Zhongguo xiu fu chong jian wai ke za zhi and Zhongguo gu shang Sichuan da xue bao. Yi xue ban is a Chinese language, as the readership of these journals is limited to Chinese people in these countries. This caused the impact factor to be low.

According to www.scimagojr.com journal grading, the majority of the publications were Q1 grade (57%), followed by Q2, Q3 and Q4 (14%, 9% and 2%, respectively). There were still 17% of articles that were published in nonindexed journals.

The article with the most citations (107 citations) was a cohort study with the following title: A prospective randomized study comparing arthroscopic single-bundle and double-bundle posterior cruciate ligament reconstructions preserving remnant fibers (doi:10.1177/0363546510382206). Among the top ten articles with the most citations, most were cohort studies (50%), followed by cross-sectional studies, surgical techniques and randomized control trials (20%, 20% and 10%, respectively) (Table 3).

This study indicates the need for collaboration research in the region. To bridge the PCL research gap among Asian countries, international collaborations with guidance from more established research centers should be encouraged.

This systematic review only focused on studies performed in Asian countries. Many researchers in Asian countries may have contributed data in an overseas-based research project or participated in research when they resided overseas. The purpose of this exclusion is to depict a clearer picture of local research output and quality.

PCL research output in Asia is low in quantity but high in quality publications, and the majority of publications come from South Korea, China and Japan, with most of the studies being cohort studies and case reports.

## Funding

The authors declare that this study had no funding resources.

## Availability of data and material

Data will be provided upon request.

### Provenance and peer review

Not commissioned, externally peer-reviewed.

## Declaration of competing interest

No potential conflicts of interest relevant to this article were reported.
